# Kiñit classification in Ethiopian chants, Azmaris and modern music: A new dataset and CNN benchmark

**DOI:** 10.1371/journal.pone.0284560

**Published:** 2023-04-20

**Authors:** Ephrem Afele Retta, Richard Sutcliffe, Eiad Almekhlafi, Yosef Kefyalew Enku, Eyob Alemu, Tigist Demssice Gemechu, Michael Abebe Berwo, Mustafa Mhamed, Jun Feng

**Affiliations:** 1 School of Information Science and Technology, Northwest University, Xi’an, China; 2 School of Computer Science and Electronic Engineering, University of Essex, Colchester, United Kingdom; 3 School of Telecommunications Engineering, Xidian University, Xi’an, China; 4 School of Computer Science and Technology, Xidian University, Xi’an, China; 5 School of Information and Civil Engineering, Chang’an University, Xi’an, China; 6 School of Information Science and Technology, Chang’an University, Xi’an, China; Vellore Institute of Technology: VIT University, INDIA

## Abstract

In this paper, we create EMIR, the first-ever Music Information Retrieval dataset for Ethiopian music. EMIR is freely available for research purposes and contains 600 sample recordings of Orthodox Tewahedo chants, traditional Azmari songs and contemporary Ethiopian secular music. Each sample is classified by five expert judges into one of four well-known Ethiopian Kiñits, Tizita, Bati, Ambassel and Anchihoye. Each Kiñit uses its own pentatonic scale and also has its own stylistic characteristics. Thus, Kiñit classification needs to combine scale identification with genre recognition. After describing the dataset, we present the Ethio Kiñits Model (EKM), based on VGG, for classifying the EMIR clips. In Experiment 1, we investigated whether Filterbank, Mel-spectrogram, Chroma, or Mel-frequency Cepstral coefficient (MFCC) features work best for Kiñit classification using EKM. MFCC was found to be superior and was therefore adopted for Experiment 2, where the performance of EKM models using MFCC was compared using three different audio sample lengths. 3s length gave the best results. In Experiment 3, EKM and four existing models were compared on the EMIR dataset: AlexNet, ResNet50, VGG16 and LSTM. EKM was found to have the best accuracy (95.00%) as well as the fastest training time. However, the performance of VGG16 (93.00%) was found not to be significantly worse (*P* < 0.01). We hope this work will encourage others to explore Ethiopian music and to experiment with other models for Kiñit classification.

## 1 Introduction

Music is an important part of everyday life. Around the world, it exists in many different forms and styles. Because musical preferences vary from person to person, categorizing music and making recommendations to listeners has become an important research topic [1] with many applications in listening apps and other platforms [2]. Multimedia file production and sharing through different mediums is increasing enormously. In consequence, indexing, browsing, and retrieval of music files has become challenging and time-consuming. Numerous digital music classification techniques have been introduced [3, 4], but the majority of them are only developed and tested on well-known Western music datasets. In Ethiopia, music classification is still being performed by individual music experts for archival or related purposes. Because of the amount of Ethiopian music now available in digital form, classification cannot be carried out with sufficient speed. As a result, even though the composer Saint Yared flourished in Ethiopia during the 6th Century [5] (p71), some five hundred years before Hildegard of Bingen [6], the music of this country is not well known elsewhere. In Ethiopia, music is based around several types of scale. Among these, four pentatonic scales (Kiñits) are particularly important [7, 8]: Tizita, Bati, Ambassel, and Anchihoye. Because the music written in each Kiñit has its own characteristic style and features, the task of Kiñit classification is closely related to that of genre classification in European music. A major challenge for Ethiopian Kiñit classification is the absence of training data. We have addressed this by creating the Ethiopian Music Information Retrieval (EMIR) dataset which includes data for the four main Kiñits. We have also developed the Ethio Kiñits Model (EKM), a genre classification model based on the well-known VGG architecture. We then carried out three experiments. The first experiment selected an appropriate method from the FilterBank, Mel-spectrogram (MelSpec), Chroma, and Mel-frequency Cepstral Coefficient (MFCC) technologies for extracting features from recordings in our EMIR dataset. MFCC was found to be the most effective in terms of accuracy and training time. The second experiment measured the effectiveness of different sample lengths for genre classification, in order to find the optimal length. The third experiment compared the classification performance of EKM and four other popular models using MFCC features, working with EMIR datasets.

The contributions of this paper are as follows:

- We create for the very first time a dataset for Ethiopian music scales (Kiñits), called EMIR. There are 600 music samples, 162 Tizita, 144 Bati, 147 Ambassel, and 147 Anchihoye.
- Five judges evaluate the recordings, and agreement between them is high (Fleiss kappa = 0.85). So the data is of high quality.
- We develop a high-performing variant of the VGG MIR model which has just four CNN layers. We call this EKM.
- We compare Filterbank, MelSpec, Chroma and Mel-frequency Cepstral Coefficient (MFCC) features and show experimentally in an MIR task that MFCC leads to higher accuracy, using the proposed EKM model and our EMIR data.
- We compare the performance of EKM using different audio sample lengths, namely one second, three seconds and five seconds, working with EMIR data. Three seconds results in the best performance.
- We apply EKM and four other architectural models to the MIR task, working with EMIR, and show that EKM is very effective and the fastest to train.

The rest of this paper is organized as follows: Section 2 reviews previous work on music genre classification. Section 3 presents the EMIR dataset, describing the rationale behind its design and the method by which it was created. Section 4 discusses feature extraction for MIR, briefly outlining Filterbank, MelSpec, Chroma and MFCC. Section 5 describes the methodology, EKM architecture and settings used for our experiments. Section 6, presents the experiments and results. Finally, Section 7 gives conclusions and next steps.

## 2 Previous work on music genre classification

According to Tzanetakis and Cook [3] in their landmark article, genres are categorial labels which classify pieces of music, based on instrumentation, rhythmic structure and harmonic content. The authors deduced three essential features for musical content, namely timbral texture, rhythm, and pitch content for Western music in various styles, including classical, jazz, pop and rock. This work paved the way for further research in the area of genre classification. Either whole recordings or homogeneous sections within them were used, and classification accuracy of 61% was achieved for ten genres, using statistical pattern recognition classifiers. The results closely matched those reported for human genre classification.

Jothilakshmi [9] applied a Gaussian mixture model (GMM), and a K-Nearest Neighbor (KNN) algorithm with spectral shape and perceptual features to Indian music datasets containing five genres. GMM gave the best accuracy with 91.25%. Rajesh [10] again utilized KNN and support vector machines (SVM), using different feature combinations. The highest classification recorded (96.05%) was by SVM using fractional MFCC with the addition of spectral roll off, flux, skewness, and kurtosis.

Al Mamun [11] used both deep learning and machine learning approaches on Bangla music datasets with six genres. The neural network model performed best compared to the machine learning methods, with accuracy 74%. Folorunso [12] investigated KNN, SVM, eXtreme Gradient Boosting (XGBoost) and Random Forest on Nigerian songs (in the ORIN dataset) with five genres. The XGBoost classifier had the highest accuracy (81.94%). De Sousa [13] implemented SVMs on a Brazilian Music Dataset (BMD) with seven genres. The set of features they proposed was specifically tailored to genre recognition yielding a high classification accuracy of 86.11%.

Kızrak [14] used Deep Belief Networks (DBNs) to classify the music genre of Turkish classical music Makams, working with seven Makam datasets. Mel Frequency Cepstral Coefficients (MFCC) were employed on the collection of features, resulting in a classification accuracy of 93.10%. Thomas and Alexander [15] used a parallel Convolutional Neural Network (CNN) to identify the mood and genre of a song. They employed Mel-Spectograms which were extracted from audio recordings, and applied a CNN to accomplish their desired task. Ali and Siddiqui [16] implemented a machine-learning algorithm to classify music genres, using KNN and SVM. To obtain information from individual songs they extracted MFCCs from audio files. Panteli et al. [17] used MFCC features and traditional machine learning to analyse recordings of world music from many countries with the aim of identifying those which are distinct. Phan et al. [18] carried out music classification in terms of environmental sound, audio scene and genre. They used four CRNN models, incorporating Mel, Gammatone, CQT and Raw inputs. The outputs were combined to produce the classification. Ma et al. [19] aimed to predict the genre of a film using Music Information Retrieval analysis. Various music features were used as input to several classifiers, including neural networks. MFCC and tonal features were found to be the best predictors of genre.

Overall, we can see that two factors need to be considered in genre classification. Firstly, appropriate features need to be extracted from the sound signal, to use in subsequent processing. Secondly, a classification model needs to be selected, to work over these features. Therefore, in our Kiñit classification work, we decided to experiment with four types of features and four well-known models, as will be described later.

## 3 Design of EMIR

### 3.1 Outline

The Ethiopian Music Information Retrieval dataset contains samples of the four main Ethiopian pentatonic scales (Kiñits): Tizita, Bati, Ambassel, and Anchihoye. Spiritual and secular songs based on these scales were collected and each was assigned to its most appropriate scale, by experts on Yared music. As previously noted, music in each scale also has distinctive stylistic characteristics, so Kiñit identification is related to genre classification for other forms of music. Classification accuracy was measured by inter-annotator agreement. Finally, music recordings were labeled and grouped together to form the EMIR dataset.

### 3.2 Recordings

There are three types of recording in EMIR: Firstly, Ethiopian Orthodox Tewahedo chants, which form part of a religious tradition dating back to the time of Saint Yared, secondly traditional Ethiopian Azmaris (songs), and thirdly modern secular Ethiopian music.

The Orthodox chants were collected from online sources such as YouTube and DireTube. Some Azmaris were specially recorded in Addis Ababa by an ethnomusicologist specialising in Azmari houses; these are traditional venues where Azmaris are studied and performed. Firstly, five typical Azmari houses were selected for the study. Secondly, these were visited on multiple occasions. Each time, a singer was asked whether they would record an Azmari of their choice which was in a specified Kiñit. If the singer knew an Azmari in that Kiñit and they agreed to the recording, it went ahead. Otherwise, another singer was asked. In this way, over several visits to each house, a collection of Azmaris in the different Kiñits was built up.

The Azmaris were recorded with an AKG Pro P4 Dynamic microphone, at a distance of 25 cm from the singer’s mouth. The audio file was saved at a 16 kHz sampling rate and 16 bits, resulting in a mono .wav file. We used the Audacity audio editing software [20] to reduce the background noise of the music signal.

Further Azmaris were collected from online sources such as YouTube etc. Finally, the secular music was collected from online sources. The breakdown of recordings can be seen in Table 1. In all cases, music clips in EMIR are limited to 30 seconds length in order to protect the copyright of the originals.

**Table 1:** Breakdown of the EMIR dataset.

Type	Source	Kiñit (Genre)				Total
		Tizita	Bati	Ambassel	Anchihoye	
Ethiopian Orthodox Tewahedo chants accompanied by traditional instruments	YouTube, DireTube	10	6	12	5	33
Songs performed in Azmari houses accompanied by traditional instruments	Recorded by musicologists	7	8	11	4	30
Azmari Songs	YouTube, DireTube	5	2	6	3	16
Secular Music	YouTube, DireTube	140	128	118	135	521
**Total**		162	144	147	147	600

### 3.3 Judgements

Five judges participated. Two of them were Ethiopian postgraduate students of Computer Science at Xidian University. Three further judges were from the Yared music school in Addis Ababa, Ethiopia. All five were experts on Yared music. Judges were responsible for the quality control of the dataset.

Each Judge listened independently to all the recordings. For each one, they either assigned it to one of the four Kiñits, or rejected it as not clearly falling into any one of them. If three or more judges assigned a recording to the same category, it was accepted for EMIR. Otherwise it was rejected.

Since we had five judges, the Fleiss kappa [21] coefficient was used to calculate the pairing agreement between participants:

$$\begin{array}{c} \kappa =\frac{{\bar{p}}_{0}-{\bar{p}}_{e}}{1-{\bar{p}}_{e}} \end{array}$$ (1)

The factor $1-{\bar{p}}_{e}$ gives the degree of agreement that is attainable above chance, and ${\bar{p}}_{0}-{\bar{p}}_{e}$ gives the degree of agreement actually achieved above chance: *k* = 1, if all the raters are in complete agreement. Evaluation of the inter-rater agreement for our dataset in terms of Fleiss kappa is 0.85. This value shows a high agreement level among our five raters.

### 3.4 Files and labeling

The tracks are all 16 KHz Mono 16-bit audio files in .wav format. Each file was labeled in the form ‘Bati1.Wav’. The first part of the name indicates the Kiñit (Tizita, Bati, Ambassel or Anchihoye); the second part indicates the number of the recording within that Kiñit (1, 2, 3…). Subsequently, recordings were stored in four different folders in the dataset.

We aimed to collect 1,000 recordings. However, because of the judgement process described in the previous section, 400 recordings were rejected because they could not be clearly assigned by judges to one Kiñit. As, for simplicity and practicality, we wished to assign each recording to exactly one class (rather than assigning them to a probability distribution over classes), we were not able to incorporate those 400 recordings. As a result, the final dataset contains 600: 162 Tizita, 144 Bati, 147 Ambassel and 147 Anchihoye (Table 1).

EMIR was split into training, validation and testing sets randomly. The training set contains 70% of the whole dataset, the validation set contains 10% and the testing set 20%. Because of the random sampling, the distribution of Kiñit types in the three subsets is very similar to that of the whole. The EMIR dataset is freely available for research purposes at https://github.com/Ethio2021/EMIR_Dataset_V1/.

## 4 Feature extraction

The main goal of the feature extraction step is to compute a sequence of feature vectors, providing a compact representation of the given input signal. In music classification, feature extraction requires much attention because classification performance depends heavily on it.

As we saw in the literature review above, genre classification involves the selection of audio features and the design of a model. In previous studies on music genre classification, four feature types have been employed: FilterBanks [22, 23], Mel-spectrograms (MelSpec) [24–27], Chroma [28–30], and MFCC [31–34]. Therefore, we used all four in our experiments to determine which would perform better for EMIR data.

### 4.1 FilterBanks

A Mel FilterBank is a triangular filter bank that works similarly to the human ear’s perception of sound; thus it is more discriminative at lower frequencies and less discriminative at higher frequencies. Mel FilterBanks are used to provide a better resolution at low frequencies and less resolution at high frequencies [35].

### 4.2 Mel-Spectrograms (MelSpec)

The signal is separated into frames and a Fast Fourier transform (FFT) is calculated for each frame. A Mel-scale is then created, where the entire frequency spectrum is separated into evenly spaced bands. A spectrogram is then created where, for each frame, the signal magnitude is decomposed into its components, corresponding to the frequencies in the Mel-scale.

### 4.3 Chroma

The chroma feature is widely used in Music Information Retrieval [36]. It is made in accordance with the twelve-tone Equal Temperament. Because notes exactly one octave apart are perceived as very similar in music, knowing the distribution of the Chroma even without the absolute frequency (i.e. the original octave) provides important musical information about the audio, and may even show perceived musical similarities not visible in the original spectra. Chroma features are usually represented as a 12-dimensional vector *v* = [*V*(1), *V*(2), *V*(3), …*V*(12)]; each element of the vector is connected with one element of the set C, C#, D, D#, E, F, F#, G, G#, A, A#, B, representing the local energy distribution of the audio signal at semitones represented by the 12 pitch names.

### 4.4 Mel-Frequency Cepstral Coefficients (MFCC)

Mel-frequency cepstral coefficients (MFCC) are widely employed to extract features from sound in various applications such as speech recognition [37], Music Emotion Recognition [38], and music genre classification [31–34, 39]. MFCC is designed using knowledge of the human auditory system, and is a common method for feature extraction in speech recognition. However, it can also be used with music because it simulates the function of the human ear [40]. Extracting features with MFCC involves splitting the signal into short frames and then, for each frame, calculating the periodogram estimate of the power spectrum. The Mel FilterBank is applied to the power spectra, to collect the energy for each filter. The log energies of all FilterBanks are calculated, and hence the Discrete Cosine Transform (DCT) of log FilterBank energies is determined. Finally, DCT coefficients 13–20 are saved, with the rest being removed.

### 4.5 Extraction

Extraction of features from music clips takes place on the EMIR dataset which is already partitioned into train, development and test sets. Initially, each clip is divided into specific lengths of time window with 50% overlap, i.e. 1s, 3s or 5s. Each clip in the dataset is limited to 30s length, as described in Section 2.2 (Recordings). Therefore, when we choose a particular window length, such as 3s, the number of samples extracted for an experiment will be the same for every clip. Moreover, as the music performances did not contain unintentional silences, special processing for removing silences was not needed.

A feature is then created for each window, resulting in a feature vector. A feature vector can contain FilterBank, Chroma, MelSpec or MFCC features. The feature vector for each music clip is used within the proposed EKM model for classification. MFCCs are extracted using 40 Mel-bands and 13 DCT coefficients.

## 5 Network architectures and setup

### 5.1 Existing classification architectures

As discussed earlier, most previous studies employ CNN-based models such as AlexNet, VGG or ResNet, or LSTMs for sound classification. The following is a short overview of these models.

- **AlexNet** [41] was the first CNN-based model to be used in the ImageNet competition, in 2012. AlexNet’s success launched a revolution, enabling numerous complex tasks to be solved with better performance. It has been widely used for music classification [25, 42]
- **VGG** [43, 44] networks appeared in 2014, developed by Oxford Robotics Institute. They were the first to employ considerably smaller 3 × 3 filters in each convolutional layer, furthermore combining them as a convolution sequence. MIR applications include Shi et al. [45] and Das et al. [46].
- **ResNet** [47] was launched in late 2015. This was the first time that networks having more than one hundred layers were trained. Subsequently it has been applied to music classification [46, 48].
- **LSTM** is a form of recurrent network which has been successfully used for music classification [49–51].

### 5.2 Proposed EKM architecture

As we have mentioned, VGG is one of the earliest CNN models used for signal processing. It is well known that the early CNN layers capture the general features of sounds such as wavelength, amplitude, etc., and later layers capture more specific features such as the spectrum and the cepstral coefficients of waves. This makes a VGG-style model suitable for the MIR task.

**Fig 1:**
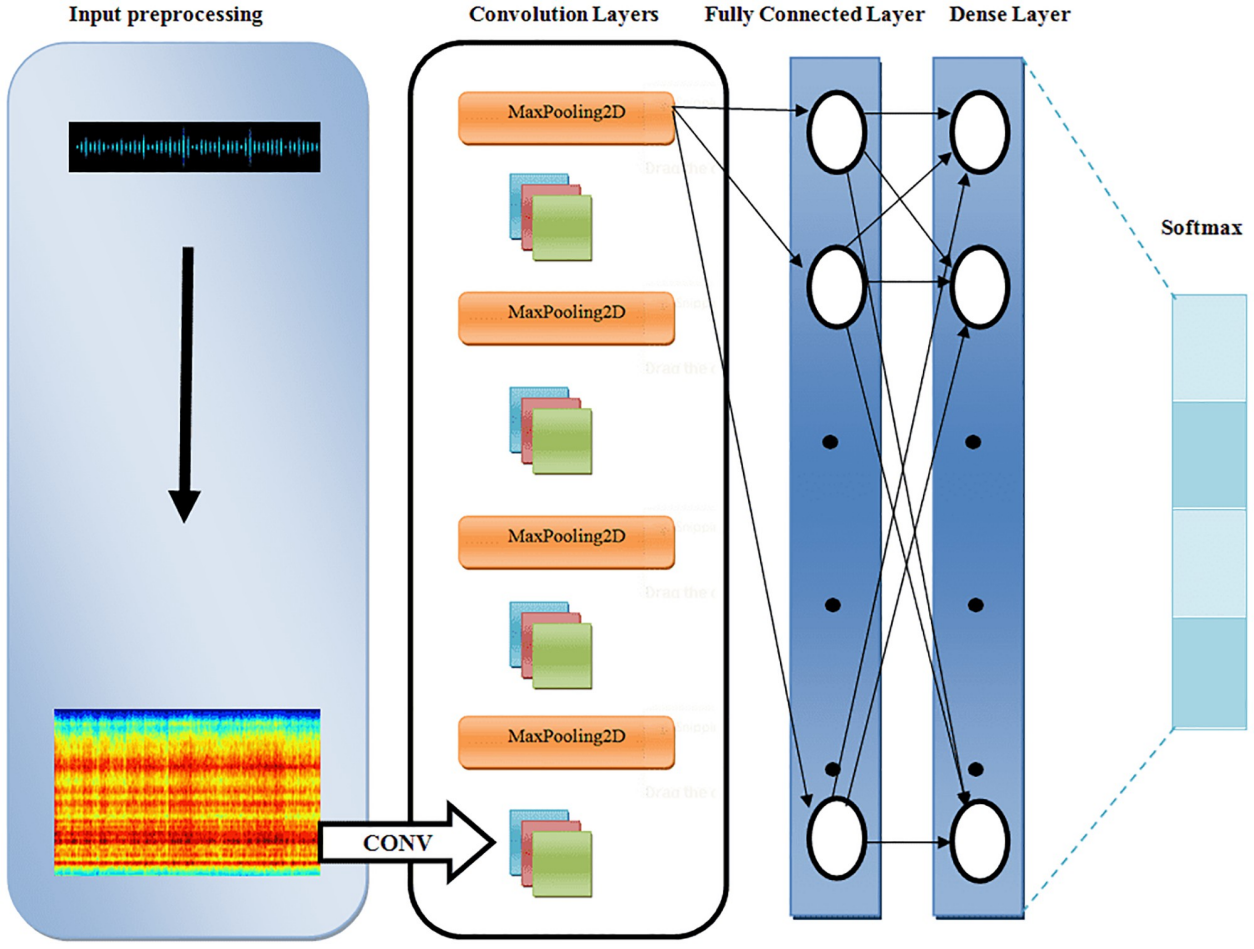
EKM architecture used in experiments.

VGG16 consists of 13 convolution layers with 3x3 kernels, 5 MaxPooling layers with pool size 2x2 filters, 2 fully connected layers, and finally a softmax layer. We therefore developed the Ethio Kiñits Model (EKM) for this work, based on VGG. After some experimentation, we found that a four-layer architecture gave the best performance. EKM thus consists of 4 convolution layers with sizes 32, 64, 128, and 256, respectively, with kernels 3x3 for the first three convolution layers and 2x2 for the last convolution layer. There are also 4 MaxPooling layers with pool size 3x3 for the first MaxPooling layer and 1x1 for the remaining layers. Finally, there is a fully connected layer and a softmax layer. The proposed model is shown in Fig 1. AlexNet, ResNet50, VGG16 and LSTM were also used for comparison.

### 5.3 Experimental setup

The standard code for AlexNet, ResNet50, VGG16 and LSTM was downloaded and used for the experiments. For EKM, the network configuration was altered (Fig 1). For the other models, the standard network configuration and parameters were used.

We extracted the FilterBank features utilizing the Python Speech Features library v0.6. MelSpec, Chroma and MFCC features were obtained with the librosa v0.7.2 library [52]. MelSpec was extracted with 128 bands, Chroma with 12 bands and MFCC with 40 bands, according to the standard settings of the tool. The model was trained in four forms, using just FilterBank, just MelSpec, just Chroma and just MFCC features, respectively.

We used the Keras deep learning library (version 2.0), with Tensorflow 1.6.0 backend, to build the classification models. The models were trained using a machine with an NVIDIA GeForce GTX 1050. The Adam optimization algorithm was used, with categorical cross-entropy as the loss function; training stopped after 250 epochs, and the batch size was set to 32.

## 6 Experiments

### 6.1 Experiment 1: Choice of features

The aim of the first experiment was to choose the most efficient technique to use for extracting features from the proposed dataset. As we have discussed in Section 3, FilterBank, MelSpec, Chroma and MFCC are four feature forms that are widely used within MIR systems. We therefore wished to determine which of these was most suitable for Kiñit classification on EMIR.

VGG CNN-based models have performed very well for other music. Therefore, the proposed EKM architecture is based on VGG, as discussed earlier. Training and testing were performed with EMIR data using 3s samples. We extracted features using four methods, FilterBank with 40 bands, MelSpec with 128 bands, Chroma with 12 bands and MFCC with 40 bands. First, the model was trained and evaluated using just Filterbank features. Training and evaluation were then repeated using just MelSpec, Chroma and MFCC features.

Data was split 70% train 10% validation and 20% test. The model was trained five times and the average result was reported.

**Fig 2:**
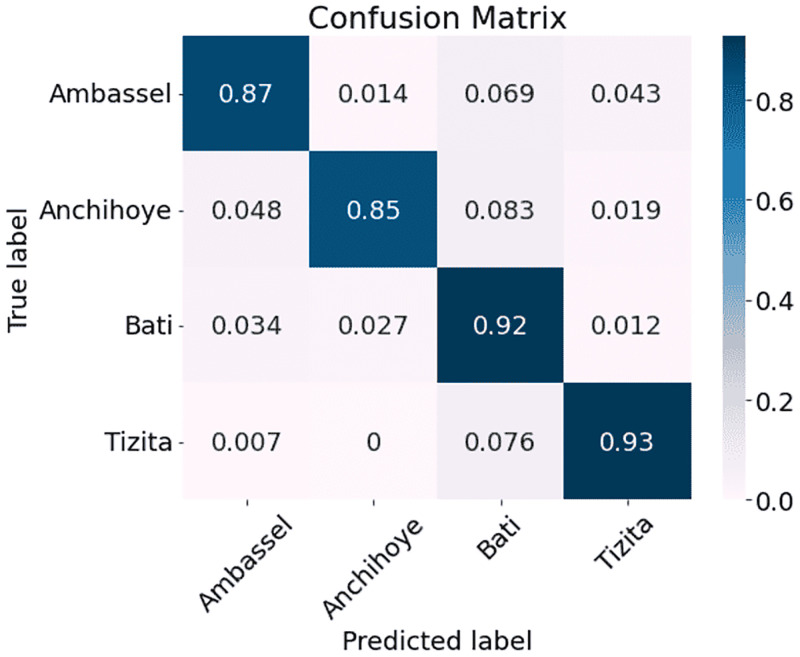
Experiment 1: EKM confusion matrices using FilterBank on EMIR.

**Fig 3:**
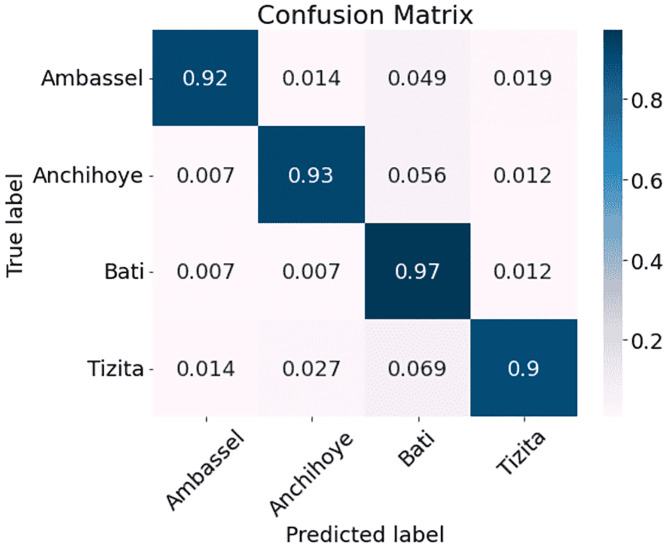
Experiment 1: EKM confusion matrices using MelSpec on EMIR.

**Fig 4:**
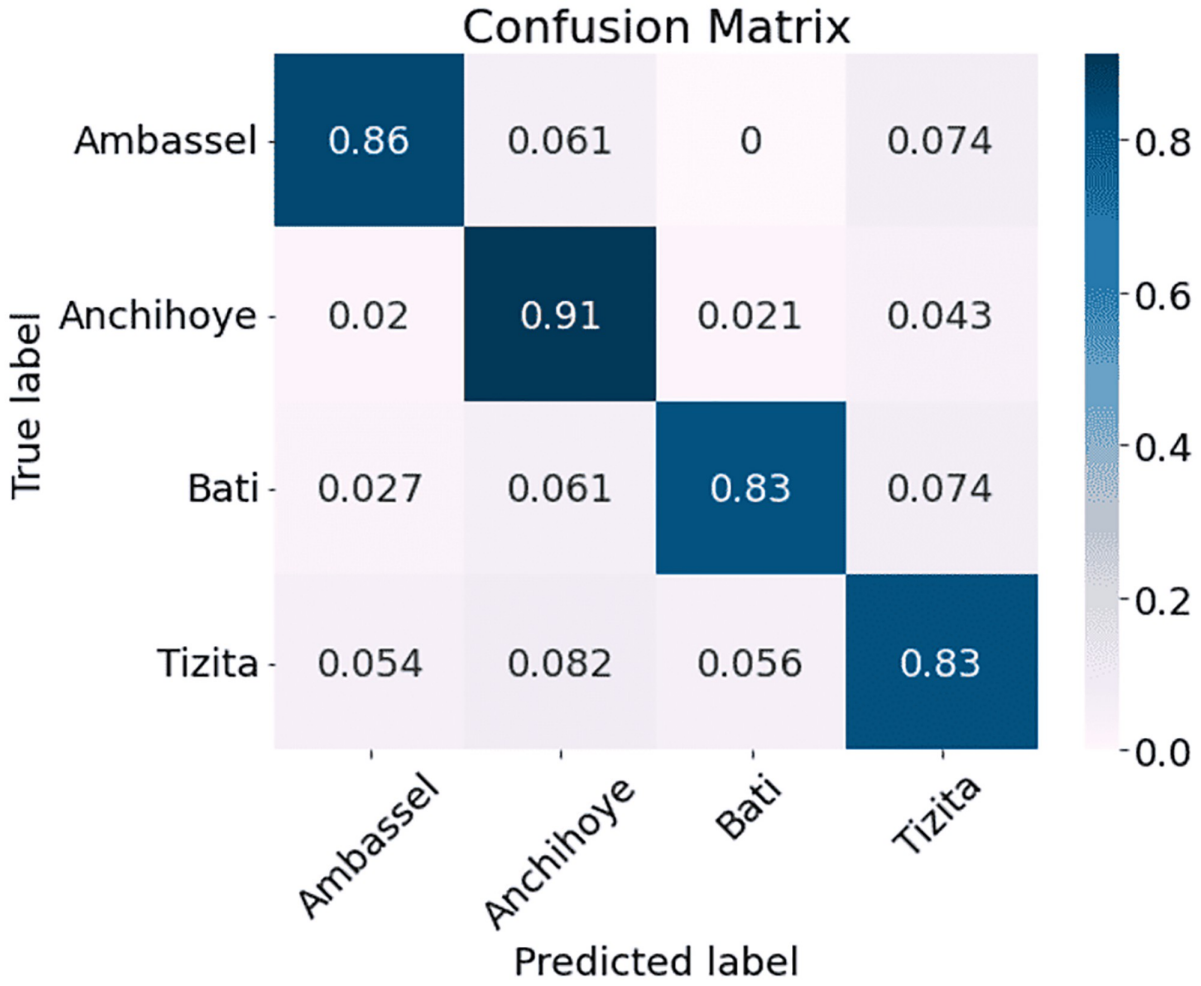
Experiment 1: EKM confusion matrices using Chroma on EMIR.

**Fig 5:**
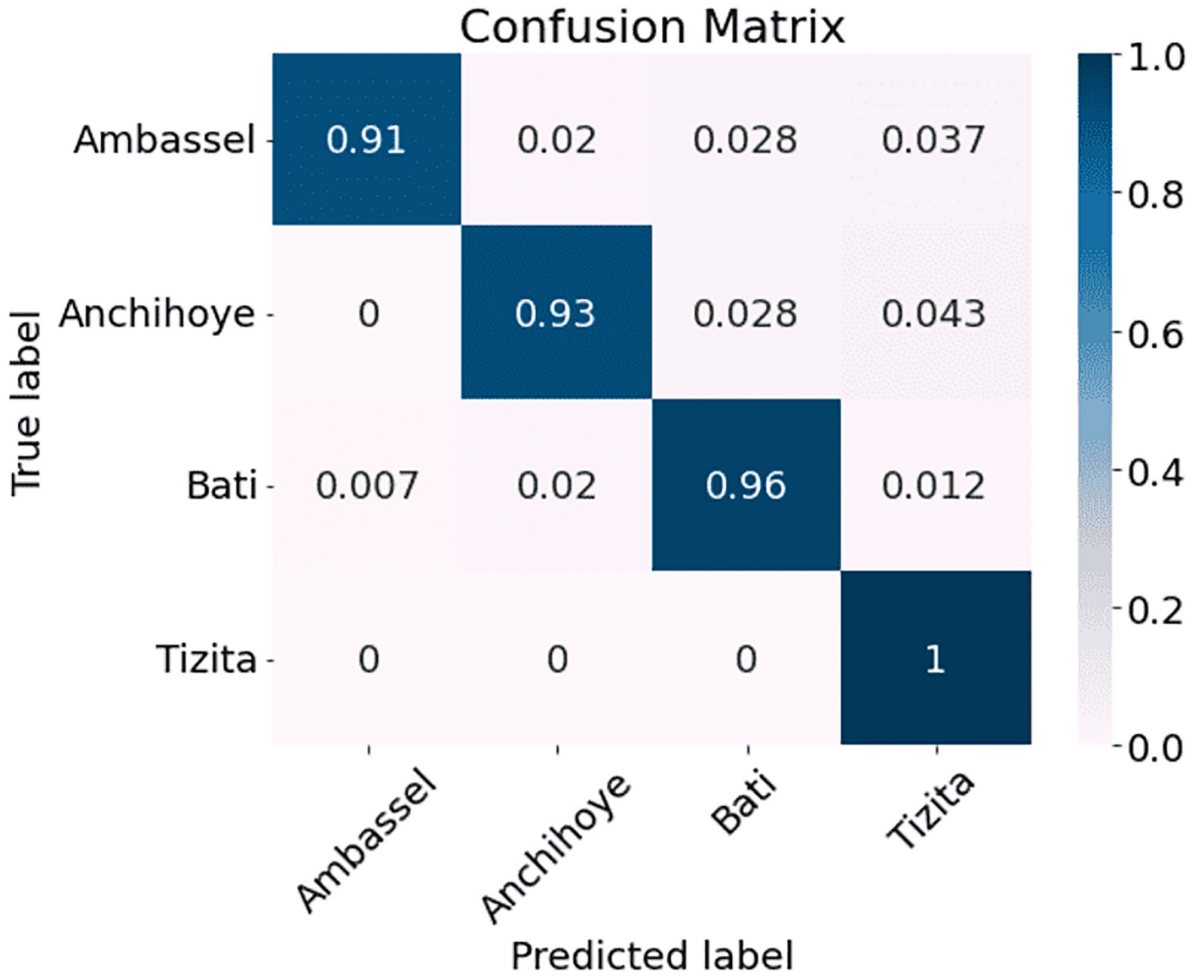
Experiment 1: EKM confusion matrices using MFCC on EMIR.

The results are presented in Table 2. As can be seen, MFCC outperforms the other three methods with a classification accuracy of 95.00% as compared with 92.83% for MelSpec, 89.33% for FilterBank, and 85.50% for Chroma. Therefore, we used MFCC processed data for subsequent experiments. After inspecting the overall results, we decided to interpret the performance on a genre level by comparing the genre classification confusion matrices arising from the model when trained with the four different types of features (Figs 2–5). The vertical axis represents the ground truth and the horizontal axis represents the prediction. The diagonal lines in all four matrices show that predictions in general reflect the ground truth. It is also clear that the network performs better with some genres than others. Figs 2–5 (FilterBank, MelSpec and MFCC) show that the Tizita, Bati, and Anchihoye scales were always easily identifiable, while Ambassel was hard to identify.

**Table 2:** Experiment 1: Recognition accuracies of VGG networks on EMIR using FilterBank, MelSpec, MFCC and Chroma features with 3s samples.

Dataset	Features				Approach
EMIR	FilterBank	MelSpec	Chroma	MFCC	EKM
	89.33%	92.83%	85.50%	95.00%	

Looking at the confusion matrices in more detail, the FilterBank EKM model mistakenly classifies 0.069 Ambassel cases as Bati and 0.043 as Tizita in Fig 2. As a result, there are only 0.87 accurate predictions for the Ambassel class. In comparison to MFCC, where 0.028 Anchihoye are projected to be Bati, FilterBank also shows lower gains in predicting 0.083 Anchihoye as Bati. Thus, Anchihoye’s correct predictions under FilterBank are 0.85 compared to those under MFCC, which are 0.93. Because MFCC can benefit from the distinction between the distributions of Bati and Tizita genres, this result appears conceivable. It is striking that the FilterBank EKM model incorrectly predicts 0.076 of the Tizita class as Bati, 0.048 of the Anchihoye as Ambassel, and 0.034 of the Bati as Ambassel.

The MelSpec, Fig 3, exhibits lower prediction improvements in predicting 0.069 Tizita as Bati. Consequently, 0.9 Tizita are correctly classified, as compared to 1.0 for MFCC. The model wrongly classifies 0.049 Ambassel and 0.056 Anchihoye both as Bati.

In Fig 4, the Chroma model wrongly classifies 0.054 and 0.056 Tizita cases as Ambassel and Bati, respectively. Additionally, it predicts 0.074 Ambassel as Tizita. So, compared to 0.91 for MFCC, only 0.86 Ambassel are correctly classified. The model inaccurately predicts 0.074 Bati as Tizita and 0.082 Tizita as Anchihoye.

When compared to the other three feature types, the use of MFCC features in Fig 5 results in significant gains for predicting the Anchihoye class (0.93 correct, vs. 0.85 for FilterBank) and the Tizita class (1.0 correct, vs. 0.9 for MelSpec). MFCC never incorrectly predicts Anchihoye as Ambassel, although FilterBank does so 0.048 times. Moreover, it predicts 0 Tizita as Bati, compared to 0.069 for MelSpec. The frequency of incorrect cases Tizita-to-Ambassel, Tizita-to-Anchihoye and Tizita-to-Bati relative to the Chroma decreased from 0.054 to 0, from 0.082 to 0 and from 0.056 to 0 respectively.

In our observation, classification performance was somewhat inconsistent across genres. While Bati, Tizita, and Anchihoye music are comparatively distinct, Ambassel is often ambiguous. This discovery is consistent with human performance, as it is more difficult for humans to identify some genres than others [9]. This also suggests that the choice of Kiñit could greatly affect the difficulty of a classification task. A dataset consisting of Tizita tracks would be significantly easier to classify, while Anchihoye would be more difficult.

### 6.2 Experiment 2: Choice of sample length

From a human perspective, it usually takes only a few seconds to determine the genre of an audio excerpt. Therefore, short samples were used, having a length of 1s, 3s or 5s. The aim of Experiment 2 was to choose the optimal sample length for the Kiñit classification of EMIR data.

Three variants of the EKM model were created, one using 1s samples for all clips, one using 3s, and one using 5s. Each model was trained five times using a 70%/10%/20% train/validation/test split, and the average results were computed.

Results are presented in Table 3. EKM had the highest accuracy on sample length 3s (95.00%), sample length 1s being close behind (94.44%). Sample length 5s was the worst (90.28%).

**Table 3:** Experiment 2: EKM model accuracy using 1s, 3s and 5s sample lengths, MFCC features and EMIR data.

Dataset	Approach	Features	Number of genres	Sample Length	Accuracy
				One second	94.44%
EMIR	EKM	MFCC	4	Three seconds	95.00%
				Five seconds	90.28%

**Fig 6:**
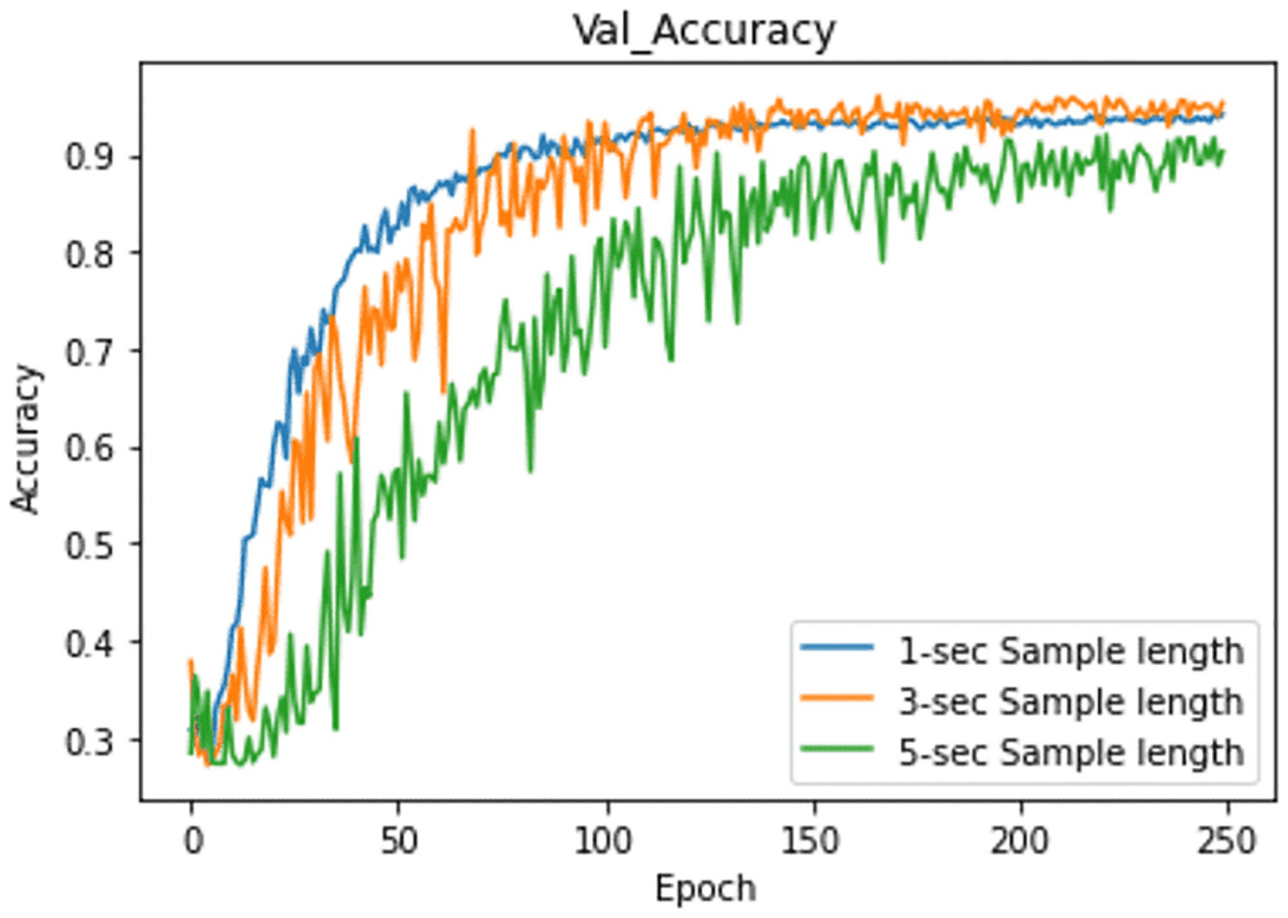
Experiment 2: Convergence curve in 250-epoch training.

Fig 6 shows the Val-accuracy curve for the three models, having sample lengths 1s, 3s and 5s. The models are trained for 250 epochs. The curves show that after the 150th epoch, the Val-accuracy starts stabilizing. The curve for three seconds looks like a better fit, while that for five seconds shows more noisy movements than the other sample lengths.

### 6.3 Experiment 3: Comparison of genre classification models

The aim was to compare four established models (Section 4) with the proposed EKM model when applied to the Kiñit classification task. Recall that the four models are AlexNet, ResNet50, VGG16 and LSTM. Once again, MFCC features were used. The network configuration for EKM was the same as in the previous Experiment (Fig 1). For the other models, the standard configuration and settings were used.

Results are presented in Table 4. As the performance of the various systems is quite similar, statistical significance testing was undertaken using the McNemar Test which Dietterich [53] recommends, in his highly cited paper, for comparing classifiers. This is a pairwise test which establishes whether two sets of results are significantly different or not. Thus, two systems (e.g. EKM and VGG16) are compared at a time. The first recording in the test set is taken and its classification by the two systems is compared to the gold standard. If it is correct for both EKM and VGG16, we assign it YesYes. If correct for EKM and incorrect for VGG16, we assign it YesNo, and so on. The process is repeated for all the recordings in the test set. A contingency table is then drawn up containing the overall counts for YesYes, YesNo, NoYes, NoNo. The table is examined to determine whether any of the counts is less than 25. If so, a modified version of the McNemar test must be used. The test is then carried out, resulting in two values, a *Test* *Statistic* and hence a *P* value. A significance level must be chosen, e.g. 0.01. If *P* < 0.01, then the difference between the two classifiers is significant, otherwise not. Finally, the process is repeated for different pairs of classifiers, e.g. EKM and ResNet50, etc. The results are shown in Table 5. In all cases, there were contingency table counts less than 25, so the exact binomial form of the test was used throughout.

**Table 4:** Experiment 3: Comparison of EKM with other CNN and LSTM models, all applied to the EMIR dataset.

No.	Model	Training Time	Accuracy
1	LSTM	00:08:46	87.50%
2	AlexNet	01:09:41	89.83%
3	ResNet50	01:37:04	90.50%
4	VGG16	01:34:09	93.00%
5	EKM	00:09:17	95.00%

**Table 5:** Statistical significance tests for Experiment 3.

Models	Test Statistic	P	Significance (*P* < 0.01)
EKM-VGG16	29.00	0.188	No
EKM-ResNet50	17.00	0.001	Yes
EKM-AlexNet	26.00	0.001	Yes
EKM-LSTM	26.00	0.000	Yes

Returning to Table 4, EKM had the highest accuracy (95.00%) with VGG16 being close behind (93.00%). However, the difference was not found to be significant (*P* < 0.01, Table 5). On the other hand, EKM was much faster than VGG16 (00:09:17 vs. 01:34:09), suggesting that it is more suitable for applying to MIR datasets. EKM was also faster than all the other models in Table 4, and all these differences were found to be significant (*P* < 0.01).

## 7 Conclusion

In this paper, we first collected what we believe to be the very first MIR dataset for Ethiopian music, working with four main pentatonic Kiñits (scales), Tizita, Bati, Ambassel and Anchihoye. We then conducted three experiments. The first experiment was to determine whether Filterbank, MelSpec, Chroma, or MFCC features were most suitable for genre classification in Ethiopian music. When used as the input to the EKM model, MFCC resulted in superior performance relative to Filterbank, MelSpec and Chroma (95.00%, 89.33%, 92.83% and 85.50%, respectively) suggesting that MFCC features are more suitable for Ethiopian music. In the second experiment, after testing several sample lengths with EKM and MFCC features, we found the optimal length to be 3s. In the third experiment, working with MFCC features and the EMIR data, we compared the performance of five different models, AlexNet, ResNet50, VGG16, LSTM, and EKM. EKM was found to have the highest accuracy (95.00%) though its superiority over VGG16 (93.00%) was not significant (*P* < 0.01). However, the training time for EKM (00:09:17) was much shorter than that for VGG16 (01:34:09).

Future work on EMIR includes enlarging the scale of the database using new elicitation techniques and studying further the effect of different genres on classification performance.
